# Plasma Medicine: Applications of Cold Atmospheric Pressure Plasma in Dermatology

**DOI:** 10.1155/2019/3873928

**Published:** 2019-09-03

**Authors:** Thoralf Bernhardt, Marie Luise Semmler, Mirijam Schäfer, Sander Bekeschus, Steffen Emmert, Lars Boeckmann

**Affiliations:** ^1^Clinic and Policlinic for Dermatology and Venereology, University Medical Center Rostock, Rostock 18057, Germany; ^2^ZIK Plasmatis, Leibniz Institute for Plasma Science and Technology (INP Greifswald), Greifswald 17489, Germany

## Abstract

The ability to produce cold plasma at atmospheric pressure conditions was the basis for the rapid growth of plasma-related application areas in biomedicine. Plasma comprises a multitude of active components such as charged particles, electric current, UV radiation, and reactive gas species which can act synergistically. Anti-itch, antimicrobial, anti-inflammatory, tissue-stimulating, blood flow-enhancing, and proapoptotic effects were demonstrated in *in vivo* and *in vitro* experiments, and until now, no resistance of pathogens against plasma treatment was observed. The combination of the different active agents and their broad range of positive effects on various diseases, especially easily accessible skin diseases, renders plasma quite attractive for applications in medicine. For medical applications, two different types of cold plasma appear suitable: indirect (plasma jet) and direct (dielectric barrier discharge—DBD) plasma sources. The DBD device PlasmaDerm® VU-2010 (CINOGY Technologies GmbH), the atmospheric pressure plasma jet (APPJ) kINPen® MED (INP Greifswald/neoplas tools GmbH), and the SteriPlas (Adtec Ltd., London, United Kingdom) are CE-certified as a medical product to treat chronic wounds in humans and showed efficacy and a good tolerability. Recently, the use of plasma in cancer research and oncology is of particular interest. Plasma has been shown to induce proapoptotic effects more efficiently in tumor cells compared with the benign counterparts, leads to cellular senescence, and—as shown *in vivo—*reduces skin tumors. To this end, a world-wide first Leibniz professorship for plasmabiotechnology in dermatology has been introduced to establish a scientific network for the investigation of the efficacy and safety of cold atmospheric plasma in dermatooncology. Hence, plasma medicine especially in dermatology holds great promise.

## 1. Plasma Medicine

Plasma medicine emerged in the last decade as an exciting new field of research at the interface between physics and the life sciences. Physical plasma can be generated by adding energy (heat or electromagnetic fields) to a neutral gas until the ionized gaseous substance becomes increasingly electrically conductive. Plasmas emit electromagnetic radiation, predominately UV radiation and visible light, and contain excited gas molecules, positively and negatively charged ions, free electrons, neutral reactive oxygen/nitrogen species (ROS/RNS), free radicals, and molecule fragments [1]. Due to its distinct characteristics compared to ordinary neutral gases, plasma is considered as a fourth state of matter (besides solid, fluid, and gaseous). In modern medicine, high-temperature plasmas are used, e.g., for sterilization of medical devices and implants [2–9]. Cold atmospheric pressure plasmas (CAP), however, can also be used for the treatment of viable tissues and thus have become a focus of medical research over the past years. Besides therapeutic applications, CAP is also used for surface modification and biological decontamination [7, 10–13].

**Figure 1:**
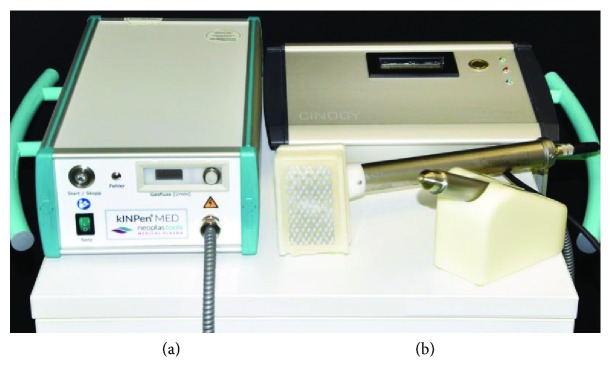
Examples of CE-certified plasma sources: (a) kINPen MED and (b) PlasmaDerm VU-2010.

A variety of different CAP devices have been developed and tested for research purposes. In general, these can be divided into direct discharge (e.g., DBD: dielectric barrier discharge) and indirect discharge (e.g., APPJ: atmospheric pressure plasma jet) devices. To date, three plasma devices have been certified for medical purposes. In 2013, the medical device kINPen® MED (INP Greifswald/neoplas tools GmbH, Greifswald, Germany), an APPJ, and PlasmaDerm® VU-2010 (CINOGY Technologies GmbH, Duderstadt, Germany), a DBD source, have been CE-certified in Germany by MEDCERT under the norm ISO 13485 (Table 1, Figure 1). Subsequently, the medical device SteriPlas (Adtec Ltd., London, United Kingdom) has been certified to be used for treatment of chronic and acute wounds, as well as for reduction of microbial load.

The use of many different devices or modifications by different research groups pose a challenge with respect to comparability of the results obtained with these devices, leading to difficulties defining long-term safety and efficacy of plasma devices in a comparable and standardized manner. Numerous organizations on a national and international level have been established to certify technical standardization. Some of these organizations are specific for certain fields such as electrical engineering or telecommunications, and others are more general. An organization for general standardization in Germany is the German Institute for Standardization (DIN). The European Committee for Standardization (CEN) and the International Organization for Standardization (ISO) provide standardization on a European and international level, respectively.

In order to establish general requirements for plasma sources in medicine, a DIN SPEC 91315 has been published by Mann and colleagues in 2014 [14]. The goal is to provide basic criteria for plasma sources to be used as medical applications. Furthermore, the efficacy of medical plasma sources as well as the safety for users (experimenters, patients, therapists, etc.) is of great importance. Although Mann et al. did not define new threshold or standard values, concerning, e.g., leakage current, UV irradiance, and formation of toxic gases, they refer to already existing standards and guidelines (DIN EN ISO 12100, DIN EN 60601-1, DIN EN 60601-1-6, and DIN EN 60601-2-57). This procedure allows a faster implementation of certified standards.

The tests described in the DIN SPEC 91315 are easy to adapt if common laboratory equipment is available, but they have to be adjusted to every plasma source with regard to individual treatment conditions. In order to obtain information on performance, effectiveness, and safety of medical plasma devices, certain physical and biological characteristics should be assessed. Physical performances include temperature, optical emission spectrometry (OES), UV irradiance, gas emission, and leakage current, and biological performances include antimicrobial activity, cytotoxicity, and chemical composition of liquid. These performances have been tested with a *μ*s-pulsed DBD source on human skin fibroblasts [15] and according to the requirements of the DIN SPEC 91315 with the plasma jet kINPen® MED [16]. Both devices meet the requirements of the DIN SPEC 91315, indicating safety and effectiveness of these devices.

Besides standardization to allow comparability of different plasma sources, a thorough risk assessment for adverse effects such as genotoxicity and mutagenicity is of tremendous importance. A couple of studies using different testing systems have shown that moderate CAP treatment does not increase genotoxicity nor mutagenicity in cultured cells [17, 18]. Although these results are promising, further studies are needed to assess the potential risk of CAP under different conditions and intensities.

As mentioned above, plasma is composed of a number of different components which all may contribute more or less to its efficacy. While the mechanisms for the efficacy of CAP are not fully understood, it is conceivable that physical components such as UV radiation or electrical current as well as chemical components such as reactive oxygen species or reactive nitrogen species play a role in the mode of action. Numerous effects of CAP such as disinfection (bacteria, fungi, and viruses), tissue regeneration (pH modulation, angiogenesis), anti-inflammation (anti-itch), and anticancerous effects (proapoptotic) have been described [19–23].

These effects provide an opportunity for CAP to be used in different applications. Applications of CAP in medicine are quite versatile and include decontamination/sterilization, use in dental medicine, enhancement of coagulation, surface coating of implants, cosmetics and plastic surgery, and treatment of skin diseases, and even the use in cancer treatment is being investigated [10, 19, 24–33]. With respect to applications in medicine, this review focuses particularly on dermatological applications of CAP (Table 2) which include treatment of atopic eczema, itch, and pain, disinfection (bacteria, parasites, and fungus), treatment of ichthyosis/epidermal barrier defect, wound healing, scar treatment, and possibly treatment of skin tumors (melanoma, squamous cell carcinoma, and basal cell carcinoma) [19–22, 25, 33].

## 2. Atopic Eczema, Itch, and Pain

A case study presented at the 20^th^ International Conference of the Society for Medical Innovation and Technology (SMIT) 2008 showed a reduction of itch for four hours and an overall reduction of itch from 8 to 3 (on a scale from 0 to 10) after daily CAP treatment for one minute of the left arm vs. basic treatment of the right arm over a period of 30 days [34]. No side effects have been observed, and overall, the eczema of this patient was reduced by two points on a scale from -5 to +5. However, a randomized two-sided placebo-controlled study on the efficacy and safety of atmospheric nonthermal argon plasma for pruritus with a total of 46 patients showed a similar improvement between plasma-treated and placebo-treated group with respect to itch [35]. In this study, patients have been treated daily for two minutes with plasma or argon only (placebo). At the end of the study, a reduction of pruritus has been observed, which was likely due to standard therapy. Another case study of a patient with chronic postoperative ear infection showed a highly significant reduction of pain after CAP application for local infection control [36]. Considering these studies, cold atmospheric plasma seems to have positive effects on atopic eczema, itch, and pain, but still, more research is necessary to confirm these effects.

## 3. Disinfection (Bacteria/Fungi/Viruses)

Several studies have elucidated the lethality of CAP on bacteria and fungi and have shown its potential as an effective tool for disinfection. A two-minute CAP treatment, for example, has been shown to be effective against a variety of bacteria including important skin and wound pathogens such as *Escherichia coli*, *group A Streptococcus*, *Methicillin-resistant Staphylococcus aureus* (MRSA), and *Pseudomonas aeruginosa*, suggesting positive effects of CAP on wound healing [12]. Furthermore, the killing of clinically relevant fungal strains by CAP has been shown *in vitro* [37]. A significant reduction of bacterial and fungal targets after plasma treatment has also been shown on model nails with onychomycosis (a fungal infection of the nail) [38]. The authors of this study conclude that the “CAP technology appears to be a safe, effective, and inexpensive therapy for fungal nail infection treatment.” Following this *in vitro* study, an *in vivo* pilot study evaluated the plasma treatment on 19 study participants with toenail onychomycosis [39]. No long-term sequelae have been observed after plasma treatment, and overall clinical cure was observed in 53.8% of participants, whereas mycological cure was observed in 15.4% of participants. A prospective randomized controlled study including 37 patients with herpes zosters (a painful skin infection caused by the varicella zoster virus) revealed that a weekday five-minute CAP treatment is safe, painless, and effective, improving initial healing of the herpes zoster lesions [40]. Taken together, promising effects of CAP have been shown in regard to disinfection with no evidence for resistance of microorganisms against the treatment. Hence, CAP provides an effective method for skin disinfection.

## 4. Ichthyosis/Epidermal Barrier Defect

An acidic protective hydrolipid film produced by perspiratory glands and sebaceous glands covers the outer layer of the skin. This hydrolipid film provides an epidermal barrier that protects the skin from drying and contains a complex microbial ecosystem, consisting of numerous bacteria [41]. The pH of the hydrolipid film is balanced between 5.4 and 5.9 in healthy skin [42]. Altered conditions in the hydrolipid film compared to healthy skin can lead to a shift in the microbial load and thus may promote disease [43, 44]. Pathogenic bacteria usually prefer pH values above 6; consequently, areas with increased pH values possess a higher susceptibility to pathogenic growth [45]. The pH values in hydrolipid films of patients with diseases such as ichthyosis or atopic dermatitis have been shown to be higher compared to pH values of healthy skin [46–48]. One reason for an increased pH in patients with atopic dermatitis may be due to a mutation in filaggrin, a protein involved in the regulation of epidermal homeostasis [49]. When human skin is treated with CAP, the hydrolipid film interacts directly with the chemical compounds of the plasma. This prompted Helmke and colleagues to investigate the effect of CAP on pH of the hydrolipid film of diseased skin [50]. Using a DBD plasma source, they treated lipid films of wool wax, pork sebum, and human lipid films with CAP and observed significant decreases in pH values. A treatment for only five seconds was sufficient to result in a decrease of pH, and treatment for 60 seconds led to a decrease of the pH down to 3.7 (from initial 4.6-6.2). The pH values of wool wax after plasma treatment remained decreased for more than two hours [50]. These studies provide first evidence for the potential treatment of epidermal barrier defects such as ichthyosis with CAP, where a decrease of the pH value would result in an inhibition of wound pathogens and, therefore, promote wound healing.

## 5. Wound Healing

**Figure 3:**
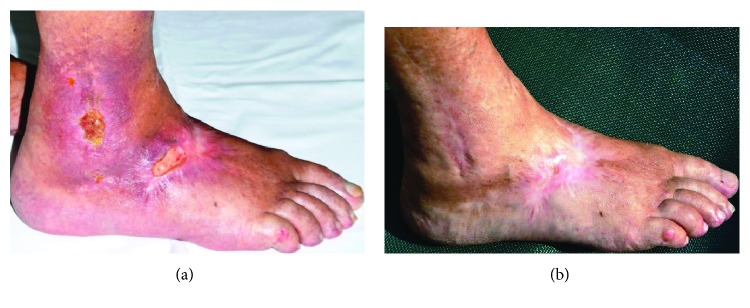
Example of successful wound healing after treatment with cold atmospheric pressure plasma (CAP): (a) chronic ulceration before CAP treatment and (b) complete healing for the first time since 14 years after CAP treatment for about 5 months.

In several clinical studies and case reports, the effect of CAP on wound healing has been assessed (Table 3). Different plasma sources have been used in these studies (e.g., kINPen MED or PlasmaDerm VU-2010, Figure 2). The use of a hand-held dielectric barrier discharge plasma generator (PlasmaDerm® VU-2010) to alleviate chronic venous leg ulcers has been assessed in a monocentric, two-armed, open, prospective, randomized, and controlled trial [51]. This pilot study included 14 patients with at least one chronic venous ulcer, which had been divided into two comparable groups each consisting of seven patients. One group received standard care, while the other group in addition to standard care also received plasma therapy. Both groups were treated three times a week for a total of eight weeks with subsequent follow-up of four weeks. While in both groups an ulcer size reduction of 50% has been observed, a greater size reduction (5.30 cm^2^ vs. 3.40 cm^2^) as well as a quicker size reduction after three weeks compared to the group without plasma treatment was found. Furthermore, there was one patient in the plasma-treated group who experienced complete healing of the ulcer. An example for successful wound healing after CAP treatment using the PlasmaDerm® VU-175 2010 is shown in Figure 3. Further prospective randomized controlled studies using a different plasma device (MicroPlaSter predecessor of SteriPlas, Adtec Healthcare) primarily aimed at decreasing the bacterial load in chronic wounds. These studies including 36 and 24 patients, respectively, showed significant reductions of bacterial load in CAP-treated chronic wounds [52, 53]. A subsequent open retrospective study with 70 patients suggests that wound healing may be accelerated by CAP treatment [36]. Improved healing of pressure ulcers was also found in a prospective randomized controlled trial including 50 patients of which 25 received CAP treatment using a Bioplasma jet device [54]. In another clinical case-control study, the efficacy of the plasma source kINPen MED was compared to octenidine treatment. This study including 16 patients with chronic leg ulcers revealed a similar reduction of bacteria using CAP treatment compared to octenidine [55]. A similar study with 34 patients showed a benefit of sequential treatment with CAP and octenidine dihydrochloride over treatment with just one of the two with respect to the antiseptic treatment [56]. In addition to studies investigating the effect of CAP on chronic wounds, a few pilot studies also looked at the efficacy of CAP on acute wounds [57–59]. Heinlin and colleagues enrolled 40 patients with skin graft donor sites on the upper leg. Equally sized areas of the wounds were assigned randomly to receive either CAP or placebo treatment [59]. From the second posttreatment day onwards, the CAP-treated sites showed significant improved healing compared to placebo treatment. In a case report study, four sterile ablative laser lesions (acute wounds) were induced in each of five volunteers [60]. The wounds received either 10 seconds, three times 10 seconds, and 30 seconds CAP or no treatment for three consecutive days. Treatment for three times 10 seconds and single treatment for 30 seconds showed best results in early stages of wound healing, and even at later stages (after six months and after one year), plasma treatment resulted in improved outcomes with respect to avoiding posttraumatic skin disorders [58]. Another study including six individuals with vacuum-generated wounds on the forearm analyzed wound healing parameters such as area decline and histomorphological characteristics [57]. Wounds were treated with either no treatment, CAP, octenidine, or sequential treatment with CAP and octenidine. A statistically significant accelerated decline was observed after CAP treatment in comparison to the other treatment groups.

Accelerated wound healing has also been observed in a mouse model treated with a cold plasma jet [61]. In cell culture experiments with HaCaT keratinocytes and MRC5 fibroblasts, an increased motility of the cells has been observed after plasma treatment, and at least in HaCaT cells, this was associated with a decreased mRNA and protein expression of Cx43. Cx43 is a gap junctional protein of keratinocytes and was shown to inhibit cell migration and wound healing [61]. These findings suggest that cold plasma enhances wound healing in chronic and slowly healing wounds.

There are several aspects of CAP that may contribute to an improved wound healing: UV radiation and reactive gas species (i.e., ozone) disinfect the wound, generation of nitric oxide (NO) or nitrogen species (NO_x_) stimulates the regeneration of tissue, and electric current stimulates angiogenesis. Furthermore, CAP leads to an acidification of the wound (decrease of pH) [12, 20, 50, 62–65].

## 6. Scar Treatment

The effect of CAP on tissue regeneration has also been elucidated for its potential in scar treatment. In one study, ten patients with acne scars received a single CAP treatment using a Plasma Skin Regeneration (PSR) system. According to patient and doctoral assessment, acne scars improved in 30% of the patients. Thermal damage to the epidermis and upper dermis had been observed for four-six days, and besides that, collagen remodeling effects without permanent pigmentary or textural irregularities as well as regenerative epidermal effects have been described [66]. Similarly, antimicrobiocidal effects of CAP and improved acne symptoms have been demonstrated using a direct DBD device. A reduction of *P. acnes* by 75% has been observed in a study with 31 volunteers [66].

Another cohort of 30 patients received weekly application of CAP for four weeks. This treatment led to a reduction of sebum production of 80%, which lasted four weeks following the treatment [66]. Although the authors concluded that their plasma source can offer a new therapeutic option for acne treatment, more studies are needed to validate these findings.

## 7. Skin Tumors

Potential applications of CAP in cancer therapy are currently explored by several research groups. The possibility of a paradigm shift in cancer therapy and the selectivity to ablate cancer cells (e.g., melanoma) while the corresponding normal cells remain unaffected have already been described in 2011 by Keidar and colleagues [19]. Detachment of SW900 cancer cells from the culture vessel after plasma treatment has been observed, whereas no detachment was observed when normal human bronchial epithelial (NHBE) cells were treated. Furthermore, one single *in vivo* treatment with CAP for two minutes of ten mice with subcutaneous bladder cancer tumors (SCaBER) showed an ablation of small tumors (about five millimeters in diameter) and a size reduction of larger tumors. While fully ablated tumors did not recur, larger tumors started to regrow but did not reach their original size even after three weeks.

Next, the *in vivo* efficacy of CAP in a murine melanoma model has been investigated. A single plasma treatment induced ablation of the tumor and decreased the growth rate markedly. This also resulted in an increased survival rate in the treatment group, with a median survival of 33.5 days compared to 24.5 days in the control group [19]. Following this study, a number of other studies assessed the efficacy of CAP as a potential therapy for cancer treatment. Daeschlein and colleagues compared the antitumor efficacy of CAP with electrochemotherapy (ECT) in a melanoma mouse model [30]. A single CAP treatment led to a significant delay of tumor growth acceleration. However, this was less effective compared to ECT, whereas the combination of CAP and ECT had the strongest effect. In light of these findings, the authors concluded that cold plasma provides a potential alternative to ECT and may serve as a new option for palliative skin melanoma therapy, either alone or in combination with ECT [30].

A murine melanoma B16/F10 cell line has been used to elucidate the effect of CAP against melanoma cells *in vitro*, showing a loss of viability of almost 100% 48 hours after CAP treatment for three minutes [29]. In addition, *in vivo* treatment with CAP of F10 cells in mice showed a decrease in growth of the tumors which was even comparable to the decrease of tumor growth achieved with chemotherapy [29].

In a clinical study with six patients suffering from locally advanced head and neck cancers, the efficacy and side effects of cold plasma treatment have been explored [67]. Two patients experienced a strong response to the treatment, resulting in a clear tumor reduction. While the tumor in one of these patients started to regrow ending with exitus letalis, the other patient was still receiving treatment, aiming for total remission. No side effects have been observed in two patients, while four patients experienced fatigue and a dry mouth. At least five of the patients had a reduction of odor, most likely due to decontamination, and four had a reduced demand of pain medication. Out of the six patients, five passed away after one to twelve months, which was not due to CAP application.

Besides ROS/RNS, the authors discussed the role of myeloid cells and the immunogenic cell death model of cancer treatment as potential mechanisms of action of CAP [67].

The potential use of CAP for cancer treatment has also been assessed *in vitro* and *in vivo* in several other nonskin cancer entities including breast cancer, pancreatic carcinomas, glioblastoma, colorectal carcinoma, and neuroblastomas [24–28]. In these studies, growth inhibition of different cell lines in culture as well as decreased tumor growth or a reduction of tumor volume has been observed in different mouse models after CAP treatment.

Although the molecular mechanisms for the efficacy of CAP on cancer cells are not fully understood, reactive oxygen species (ROS) and charged particles have been determined to be major contributors to plasma-induced cell death [68].

Taken together, these findings indicate a general efficacy of CAP against various cancer entities, suggesting plasma to be a potential new therapy against diverse cancerous diseases. However, to integrate plasma treatment into modern cancer therapy, further studies have to be conducted.

## 8. Actinic Keratosis

The precancerous actinic keratosis is a patch of thick, scaly, or crusty skin [69–71]. There are several options to treat actinic keratosis in order to prevent the development of squamous cell carcinomas. One option is the use of ingenol mebutate, which works in two ways: first, it leads to rapid lesion necrosis beginning one to two hours after application of ingenol mebutate, resulting from mitochondrial swelling and membrane disruption as well as membrane depolarization [72]. This process causes an inflammatory response, which, subsequently, leads to specific neutrophil-mediated antibody-dependent cellular cytotoxicity (ADCC) within days. These specific antibodies bind to specific antigens on dysplastic epidermal cells, as well as to receptors of infiltrating neutrophils, thereby causing the release of cytotoxic agents (e.g., ROS), which then destroy dysplastic epidermal cells [72]. The importance of the immune cell reaction against LK2 tumors (squamous cell carcinoma) has been shown in mice by Challacombe and colleagues. Following the treatment with PEP005 (ingenol-3-angelate), neutrophil-depleted mice showed a significant higher tumor volume in comparison to the control mice [73].

Similar to that, CAP seems to positively influence the healing of actinic keratosis as well. This has been shown in a pilot study with seven patients, who were treated twice a week, with CAP for two minutes, for a total of seven treatments [74]. All patients have shown promising responses with no adverse events so that further investment does not only seem necessary but also very promising. In a retrospective study of 12 patients with advanced head and neck squamous cell carcinomas, CAP was used to decontaminate infected cancer ulcerations and evaluated for its anticancer effects [75]. Analysis of data from this study showed a decreased request for pain medication, a reduction of typical fetid odor related to a reduction of microbial load, and in some cases even a superficial partial remission of tumor and wound healing of infected ulcerations.

A case report from Daeschlein and colleagues showed promising results of CAP in a patient with recalcitrant actinic keratosis of the scalp [76]. No actinic lesion relapse has been observed until at least 26 months after a single treatment with CAP, and furthermore, scar formation had been proven.

Taken together, these studies indicate a promising application of CAP as an innovative therapy for skin cancers or precancerous conditions, but the evidence is still sparse, and more basic research as well as more clinical studies are needed. To foster the investigation of the efficacy and safety of CAP in dermatooncology, a world-wide first Leibniz professorship for plasmabiotechnology in dermatology has been introduced, offering an opportunity to bring clinical needs and scientific findings together, while integrating CAP treatment in dermatology [77].

## 9. Conclusions

CAP has been shown to be a promising and inexpensive treatment for a variety of different diseases. While CAP already reached standard medical care status for wound treatment, only preliminary data for its effects in oncology are available. Continuing efforts in this emerging and highly dynamic field of plasma medicine will be necessary to further explore the full therapeutic potential of CAP and to fully understand its mechanisms of action. Whereas the development of novel plasma sources and modifications of existing devices will open up more opportunities, it will be crucial to adhere to certain standards in order to enable comparability of results obtained in different studies. Overall, the exciting field of plasma medicine is expanding and thus is providing increasing evidence for the use of CAP as a treatment option for a variety of dermatological diseases.

## Figures and Tables

**Figure 2:**
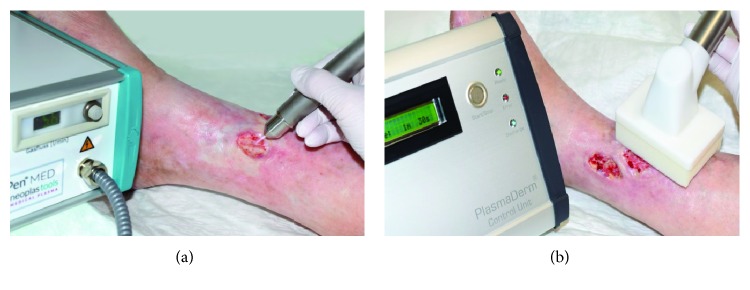
Treatment of chronic ulceration with cold atmospheric pressure plasma (CAP): (a) kINPen MED and (b) PlasmaDerm VU-2010.

**Table 1:** CE-certified plasma sources developed by systematic research and used in clinical studies.

Atmospheric pressure plasma jet kINPen® MED (INP Greifswald/neoplas tools GmbH, Greifswald, Germany)
Dielectric barrier discharge source PlasmaDerm® VU-2010 (CINOGY GmbH plasma technology for health, Duderstadt, Germany)
SteriPlas (Adtec Ltd., London, United Kingdom)

**Table 2:** Dermatological applications of cold atmospheric pressure plasma (CAP) tested in clinical pilot or case-control studies.

Atopic eczema, itch, and pain relief
Disinfection (bacteria/fungi/viruses)
Treatment of epidermal barrier defects such as ichthyosis
Wound healing
Scar treatment
Treatment of skin tumors

**Table 3:** Overview of studies on treatment of chronic and acute wounds with cold atmospheric pressure plasma (CAP).

Title	Number of subjects	Conclusion	Wound	Reference
A first prospective randomized controlled trial to decrease bacterial load using cold atmospheric argon plasma on chronic wounds in patients	36 patients	Highly significant reduction in bacterial load	Chronic	Isbary et al. [53]
Successful and safe use of 2 min cold atmospheric argon plasma in chronic wounds: results of a randomized controlled trial	24 patients	MicroPlaSter alpha: significant reduction in bacterial load MicroPlaSter beta: highly significant reduction in bacterial load	Chronic	Isbary et al. [52]
Cold atmospheric argon plasma treatment may accelerate wound healing in chronic wounds: results of an open retrospective randomized controlled study in vivo	70 patients	Wound healing may be accelerated by CAP, particularly for chronic venous ulcers	Chronic	Isbary et al. [78]
The healing effect of low-temperature atmospheric-pressure plasma in pressure ulcer: a randomized controlled trial	50 patients	CAP-treated group had significantly better PUSH (pressure ulcer scale for healing) scores and exudate amount	Chronic	Chuangsuwanich et al. [54]
Clinical use of cold atmospheric pressure argon plasma in chronic leg ulcers: a pilot study	16 patients	Immediate antimicrobial effects of CAP plasma almost comparable to octenidine without signs of cytotoxicity	Chronic	Ulrich et al. [55]
Combined antibacterial effects of tissue-tolerable plasma and a modern conventional liquid antiseptic on chronic wound treatment	34 patients	The combined use of CAP and conventional antiseptics might represent the most efficient strategy for antiseptic treatment of chronic wounds	Chronic	Klebes et al. [56]
Alleviation of chronic venous leg ulcers with a hand-held dielectric barrier discharge plasma generator (PlasmaDerm® VU-2010): results of a monocentric, two-armed, open, prospective, randomized and controlled trial	14 patients	PlasmaDerm® VU-2010 device is safe and effective in patients with chronic venous leg ulcers	Chronic	Brehmer et al. [51]
Randomized placebo-controlled human pilot study of cold atmospheric argon plasma on skin graft donor sites	40 patients	Donor site wound areas treated with plasma showed significantly improved healing compared with placebo-treated areas	Acute	Heinlin et al. [59]
Experimental recovery of CO_2_-laser skin lesions by plasma stimulation	5 experimental case reports	Nonthermal atmospheric pressure plasma stimulation of laser skin lesion recovery looks promising	Acute	Metelmann et al. [60]
Scar formation of laser skin lesions after cold atmospheric pressure plasma (CAP) treatment: a clinical long-term observation	20 laser lesions in 5 individuals	Plasma treatment seems to support the inflammation needed for tissue regeneration	Acute	Metelmann et al. [58]
Laser scanning microscopy as a means to assess the augmentation of tissue repair by exposition of wounds to tissue-tolerable plasma	6 subjects with vacuum-generated wounds	CAP led to a significantly more rapid area decline in comparison to no treatment, treatment with octenidine, and sequential treatment with CAP and octenidine	Acute	Vandersee et al. [57]
